# Mitigating SUV uncertainties using total body PET imaging

**DOI:** 10.1007/s00259-023-06503-x

**Published:** 2023-11-13

**Authors:** Charlotte L. C. Smith, Gerben J. C. Zwezerijnen, Marijke E. den Hollander, Jolijn Weijland, Maqsood Yaqub, Ronald Boellaard

**Affiliations:** 1 Department of Radiology and Nuclear Medicine, Amsterdam UMC location Vrije Universiteit Amsterdam, De Boelelaan 1117 1081 HV, Amsterdam, The Netherlands; 2 Cancer Center Amsterdam, Imaging and Biomarkers, Amsterdam, The Netherlands

**Keywords:** Image based, Body weight, Quality control, LAFOV PET, ^18^F-FDG PET/CT

## Abstract

**Purpose:**

Standardised uptake values (SUV) are commonly used to quantify ^18^F-FDG lesion uptake. However, SUVs may suffer from several uncertainties and errors. Long-axial field-of-view (LAFOV) PET/CT systems might enable image-based quality control (QC) by deriving ^18^F-FDG activity and weight from total body (TB) ^18^F-FDG PET images. In this study, we aimed to develop these image-based QC to reduce errors and mitigate SUV uncertainties.

**Methods:**

Twenty-five out of 81 patient scans from a LAFOV PET/CT system were used to determine regression fits for deriving of image-derived activity and weight. Thereafter, the regression fits were applied to 56 independent ^18^F-FDG PET scans from the same scanner to determine if injected activity and weight could be obtained accurately from TB and half-body (HB) scans. Additionally, we studied the impact of image-based values on the precision of liver SUVmean and lesion SUVpeak. Finally, 20 scans were acquired from a short-axial field-of-view (SAFOV) PET/CT system to determine if the regression fits also applied to HB scans from a SAFOV system.

**Results:**

Both TB and HB ^18^F-FDG activity and weight significantly predicted reported injected activity (*r* = 0.999; *r* = 0.984) and weight (*r* = 0.999; *r* = 0.987), respectively. After applying the regression fits, ^18^F-FDG activity and weight were accurately derived within 4.8% and 3.2% from TB scans and within 4.9% and 3.1% from HB, respectively. Image-derived values also mitigated liver and lesion SUV variability compared with reported values. Moreover, ^18^F-FDG activity and weight obtained from a SAFOV scanner were derived within 6.7% and 4.5%, respectively.

**Conclusion:**

^18^F-FDG activity and weight can be derived accurately from TB and HB scans, and image-derived values improved SUV precision and corrected for lesion SUV errors. Therefore, image-derived values should be included as QC to generate a more reliable and reproducible quantitative uptake measurement.

## Introduction

^18^F-fluoro-deoxy-glucose (^18^F-FDG) positron emission tomography–computed tomography (PET/CT) is a non-invasive imaging technique extensively used in oncology for diagnosis, response prediction, staging, and treatment monitoring [1–3]. Lesion ^18^F-FDG uptake and distributions are mainly graded by visual interpretation [4] and by semi-quantitative uptake measurements, such as standardised uptake values (SUV) [2, 4–6]. The SUV is probably the most commonly used semi-quantitative uptake measure for ^18^F-FDG PET/CT studies and measures normalised FDG uptake in tissue and tumour by normalising the amount of injected radioactive dose by the body weight [4–6].

SUV is susceptible to several biological, technical, and physical related errors [2]. On average, most factors have limited effect on SUV (< 15%). However, the accumulation of several small errors can result in substantial outcome differences [2, 7]. Harmonisation and standardisation of scan and reconstruction protocols, such as the European Association of Nuclear Medicine Research Ltd. (EARL) accreditation program, reduce the intra- and inter-institute variability which increases the reproducibility and repeatability of SUV [1, 8]. Even with these guidelines, SUV remains prone to several errors such as data entry errors, weight over- and underestimation, and incorrect clock synchronisation. Although these errors could be avoided by strictly following the recommendations presented in the aforementioned guidelines, in practice these errors still occur frequently. For example, many sites still enter body weight and injected ^18^F-FDG activity manually, and most PET units do not systematically weigh patients on a calibrated scale before scanning, as shown by Lasnon et al. [9]. In addition, approximately 3.5% of the patients cannot be weighed on a calibrated scale before the scan since they are bedridden [9]. Weighing patients prior to scanning is crucial since the course of the disease and treatment can cause a large variability in body weight. Therefore, deriving body weight and injected ^18^F-FDG activity from ^18^F-FDG PET images may generate a more reliable and reproducible metric. Moreover, an image-derived method to calculate SUV may also address missing values in the DICOM header due to rigorous anonymisation of data in multi-centre studies hampering centralised analysis and/or causing data loss.

The aim of this study is to examine if image-derived body weight and injected ^18^F-FDG activity mitigate SUV uncertainties. To achieve this, injected ^18^F-FDG activity and body weight are derived from total body (TB) ^18^F-FDG PET images obtained on a long-axial field-of-view (LAFOV) PET/CT system. The liver SUVmean and lesion SUVpeak of the hottest lesion are used to determine if the image-derived method removes errors and therefore reduces SUV variability due to these errors. Additionally, we will provide and evaluate a model to predict injected ^18^F-FDG activity and body weight from half-body (HB) ^18^F-FDG PET scans. The HB scans typically range from the skull vertex to mid-thigh [10]. Finally, we tested if the proposed method also applied to a standard short-axial field-of-view (SAFOV) PET/CT system.

## Material and methods

### Datasets

For this study, ^18^F-FDG phantoms and three different patient datasets were examined. Patient scans were collected at the Amsterdam UMC, location VUmc from ongoing clinical investigations or restaging studies [11]. The use of anonymised clinical data for technical scientific purposes was waived by the VU Medical Center ethics review board.

All scans were scanned at VUmc using either the Siemens Vision Quadra PET/CT system (Siemens Healthineers, Knoxville, TN, USA) (LAFOV PET/CT system) or the Philips Ingenuity PET/CT system (SAFOV PET/CT system). The algorithms were first evaluated using phantoms scanned on the LAFOV PET/CT system. Data from two uniform cylindrical (9.293 mL, 20-cm diameter, 3.4–4.0 kBq/mL ^18^F-FDG) and a NEMA image quality (background 1.9 kBq/mL ^18^F-FDG and spheres 18.2 kBq/mL ^18^F-FDG) phantom were included. Next, ^18^F-FDG PET/CT scans were collected from the LAFOV PET/CT system and used as a test dataset. All patients were weighed on the day of the PET/CT scan, prior to the ^18^F-FDG injection. We subtracted 2.0 kg from the measured body weight since patients were weighed with shoes and clothes on [12]. Thereafter, we randomly selected ^18^F-FDG PET/CT scans from the LAFOV PET/CT system. The scans were examined as an independent validation dataset to test the regression parameters. To validate the HB regression model, scans with inaccuracies in injected activity, body weight, or any other known SUV uncertainties summarised in Boellaard et al. [2] were excluded. Finally, ^18^F-FDG PET/CT scans from the SAFOV PET/CT system were selected to examine if the regression parameters also applied to scans generated on a SAFOV scanner.

The scans performed on the LAFOV PET/CT system were reconstructed as TB and HB scans. The TB PET scans were acquired using two static bed positions, each covering 106 cm [13]. The first bed position covered the skull vertex to mid-thigh and lasted 8 min. The second bed position lasted 2 min and covered the legs. The HB reconstructions only consisted of the first bed position. The scans included from the SAFOV PET/CT system were all HB scans ranging from the skull vertex to mid-thigh. The amount of bed positions depended on the length of the patient. All scans were performed according to the recommendations provided in the EANM guidelines [1]. In short, patients had a fasting status of 4–6 h before tracer administration, plasma glucose levels < 11.0 mmol/L, and a standard uptake time of 55–65 min was applied [1]. Patients received between 1.5 and 3.0 MBq/kg radioactive ^18^F-FDG by an intravenous bolus injection. The scans were reconstructed applying an EARL2-compliant protocol to obtain images adhering to the European guidelines for multi-centre PET image quantification and harmonisation [1]. The LAFOV PET scans (for each bed position) were reconstructed with the EARL2-compliant reconstruction protocol consisting of 3D OSEM with 5 subsets, 4 iterations, matrix size of 220 × 220, 531-533 slices in the z-dimension, a voxel size of 3.2 × 3.2 × 2 mm^3^, point spread function (PSF), time of flight (ToF), and 4-mm Gaussian filter [14, 15]. The SAFOV PET scans (for each bed position) were reconstructed with the EARL2-compliant reconstruction protocol consisting of BLOB-OS-TF with 21 subsets, 3 iterations, resolution recovery, matrix size of 144 × 144 and a voxel size of 4 × 4 × 4 mm^3^. There were no additional filters applied [11].

### Image analysis

For the three repeated phantom experiments, the EARL2 analysis procedure was followed to verify the accuracy of scanner calibration and thus the accuracy of deriving the actual activity in the phantoms compared to image-based activity.

Next, injected ^18^F-FDG activity and patient weight were derived from the ^18^F-FDG PET images. The injected ^18^F-FDG activity was derived by counting the total image activity in all voxels while applying a correction for radioactive decay toward injection time to obtain an estimate for injected activity. Body weight was calculated by deriving the patient volume from all voxels with ^18^F-FDG activity higher than zero and taking the mean density of 1.0 g/mL. TB image-derived body weight and ^18^F-FDG activity from the test dataset were used in a linear model to predict reported body weight and injected activity. The HB image-derived values were combined with reported patient length in a multi-linear model to predict report weight and injected activity.

To validate the regression parameters, ^18^F-FDG PET scans from the LAFOV PET/CT system were randomly selected and used as an independent validation dataset. Both TB and HB image-derived body weight and ^18^F-FDG activity were corrected with the regression fits. The data was tested to determine if the corrected image-derived values agreed with the reported values. In addition, the validation dataset was used to study if image-derived values mitigated liver SUVmean and lesion SUVpeak variability by resolving errors. Liver uptake was analysed with a spherical volume-of-interest (VOI) of 3-cm diameter placed in the (unaffected) right upper lobe [1, 16]. Lesion uptake was examined using the SUVpeak (1.0 mL spherical VOI positioned within the tumour to yield the highest average peak value [17]) for the hottest lesion. The hottest lesion was selected with a semi-automated analysis software tool Accurate (developed in IDL version 8.4 (Harris Geospatial Solutions, Bloomfield, USA)) [18]. An SUV above 4.0 was used as a threshold for lesion contouring. Liver and lesion SUVs were derived from the selected VOIs and calculated based on (1) reported injected activity and body weight, (2) image-derived ^18^F-FDG activity and reported body weight, (3) reported injected activity and image-derived body weight, and (4) image-derived ^18^F-FDG activity and body weight. This was done for both the TB and HB image-derived values.

Finally, ^18^F-FDG PET scans from a SAFOV PET/CT system were used to examine if the HB regression fits also applied to ^18^F-FDG PET scans generated on a SAFOV PET/CT system. Body weight and ^18^F-FDG activity were derived from the HB PET images and corrected by applying the multi-linear regression models. The corrected image-derived body weight and ^18^F-FDG activity were tested on whether these were consistent with reported body weight and injected ^18^F-FDG activity.

### Statistical analysis

Statistical analyses and data presentation were performed in R (version 4.2.2). Estimation of injected activity and patient weight from image-derived ^18^F-FDG activity and body weight was based on a linear regression analysis through the origin. The injected activity was calculated according to Eq. 1:

1 $${\mathrm{Act}}^{\mathrm{INJ}} = a \times {\mathrm{Act}}^{\mathrm{IMG}}$$

where Act^INJ^ is the reported net injected activity (MBq) and Act^IMG^ is the total image-derived ^18^F-FDG activity (MBq). Patient body weight was generated using Eq. 2:

2 $${\mathrm{W}}^{\mathrm{PAT}} = a \times {W}^{\mathrm{IMG}}$$

where *W*^PAT^ is the reported body weight (kg) of the patient and W^IMG^ is the image-derived body weight (kg). Reported injected activity and body weight were also predicted from HB image-derived ^18^F-FDG activity and body weight together with reported patient length. Reported injected activity or body weight were the quantitative dependent variables, and HB image-derived ^18^F-FDG activity or body weight together with reported patient length were the independent variables. The injected activity was calculated according to Eq. 3:

3 $${\mathrm{Act}}^{\mathrm{INJ}} = a + b \times {\mathrm{Act}}^{\mathrm{HB}-\mathrm{IMG}} + c \times L$$

where Act^HB-IMG^ is the HB image-derived ^18^F-FDG activity (MBq) and *L* the reported length (cm) of the patient. Act^INJ^ is still the reported net injected activity (MBq). Patient body weight was determined according to Eq. 4:

4 $${W}^{\mathrm{PAT}} = a + b \times {W}^{\mathrm{HB}-\mathrm{IMG}} + c \times \mathrm{ L}$$

where *W*^HB-IMG^ is the HB image-derived body weight (kg). *W*^PAT^ is still the reported body weight (kg), and *L* reported length (cm). Statistically signification associations (*p* ≤ 0.050) were analysed with a Pearson correlation or Spearman’s rank-order correlation test, when appropriate. Lesion SUVpeak and liver SUVmean were described using median values and interquartile ranges (IQR) and presented using Violin plots, including Tukey’s boxplots.

## Results

Three phantoms were scanned on the LAFOV PET/CT system. For all three phantoms, differences between image-derived and injected activity agreed within 5.8% (5.8% and 2.7% for the uniform cylinders and −1.7% for the NEMA image quality PET phantom). The relative absolute difference between image-derived and injected activity for the three phantoms was 3.4% ± 2.1%.

### Test dataset

For the test dataset, *n* = 25 ^18^F-FDG PET scans from the LAFOV PET/CT system were included. We found that image-derived ^18^F-FDG activity significantly predicted reported injected activity (*r* = 0.999, *p* < 0.0001). The coefficient of Eq. 1 was equal to *a* = 1.14. Image-derived patient weight also significantly predicted reported weight (*r* = 0.999, *p* < 0.0001), where the coefficient of Eq. 2 was equal to *a* = 1.03. Adding the regression coefficient to Eqs. 1 and 2 resulted in the following equations:

5 $${\mathrm{Act}}^{\mathrm{INJ}} = 1.14 \times {\mathrm{Act}}^{\mathrm{IMG}}$$

6 $${W}^{\mathrm{PAT}}= 1.03 \times {W}^{\mathrm{IMG}}$$

Overall, HB image-derived ^18^F-FDG activity together with reported length significantly predicted injected activity (*r* = 0.984, *p* < 0.0001). HB image-derived ^18^F-FDG activity significantly predicted injected activity (*p* < 0.0001), however, reported patient length did not significantly predict injected activity (*p* = 0.225). The multiple linear regression showed in Eq. 3 had an offset of *a* = −35.01. The coefficients of Eq. 3 were equal to *b* = 1.26 and *c* = 0.21. In addition, HB image-derived patient weight and reported length significantly predicted reported body weight (*r* = 0.987, *p* < 0.0001). Both HB image-derived body weight and reported length significantly predicted reported weight (*p* < 0.0001). The offset of Eq. 4 was equal to *a* = −71.33 and the coefficients were equal to *b* = 1.10 and *c* = 0.47. Adding the regression coefficients and offset to Eqs. 3 and 4 resulted in the following equations:

7 $${\mathrm{Act}}^{\mathrm{INJ}} = -35.01 + 1.26 \times {\mathrm{Act}}^{\mathrm{HB}-\mathrm{IMG}} + 0.21 \times L$$

8 $${W}^{\mathrm{PAT}} = -71.33 + 1.10 \times {W}^{\mathrm{HB}-\mathrm{IMG}} + 0.47 \times L$$

### Validation dataset

**Fig. 1:**
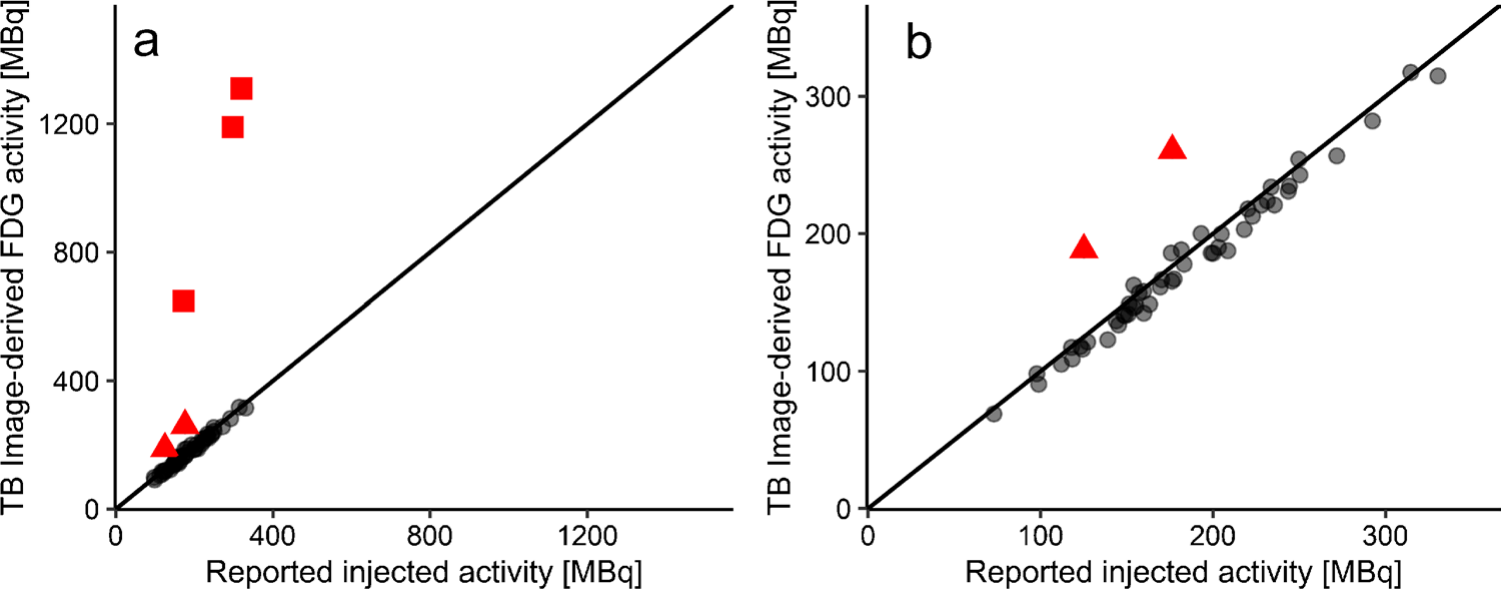
The correlation between reported injected activity and image-derived ^18^F-FDG activity (MBq) derived from total body (TB) ^18^F-FDG PET images acquired on a long-axial field-of-view (LAFOV) PET/CT system (**a**) and a zoomed in display of the correlation (**b**). The black line is the line of identity (LoI), the red squares indicate an error due to selecting a different isotope, and the red triangles indicate errors due to incorrect clock synchronisation

**Fig. 2:**
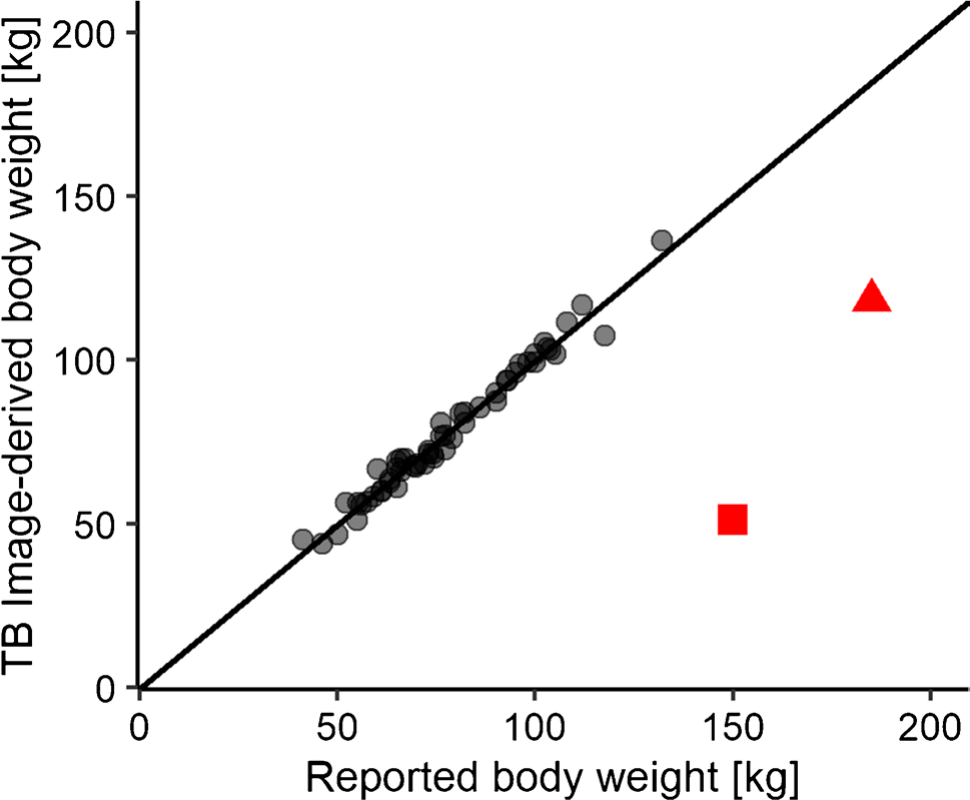
The correlation between reported and image-derived body weight (kg) derived from total body (TB) ^18^F-FDG PET images acquired on a long-axial field-of-view (LAFOV) PET/CT system. The black line is the line of identity (LoI), the red square indicate an error due to a data entry error in weight, and the red triangle indicate an error due to switching reported length and weight

Fifty-six ^18^F-FDG PET scans were randomly selected from the LAFOV PET/CT system to validate the aforementioned regression parameters. After applying the obtained regression fits, we found a strong correlation between TB image-derived ^18^F-FDG activity and reported injected activity (*r* = 0.928, *p* < 0.0001), illustrated in Fig. 1a, b. Five data points (illustrated in red in Fig. 1a) were considered errors after data analysis due to selecting the wrong isotope (red square) or clock differences due to the switch from summer to winter time (red triangle). After the exclusion of these five data points, the relative absolute difference between reported injected activity and image-derived ^18^F-FDG activity was 4.8% ± 2.7%. TB image-derived body weight correlated strongly with reported body weight (*r* = 0.899, *p* < 0.0001), illustrated in Fig. 2. Two data points (illustrated in red in Fig. 2) were considered errors due to a data entry error in weight (red square) or due to switching reported length and weight with each other (red triangle). After excluding the two data points, the relative absolute difference between reported and image-derived body weight was 3.2% ± 2.7%.

**Fig. 3:**
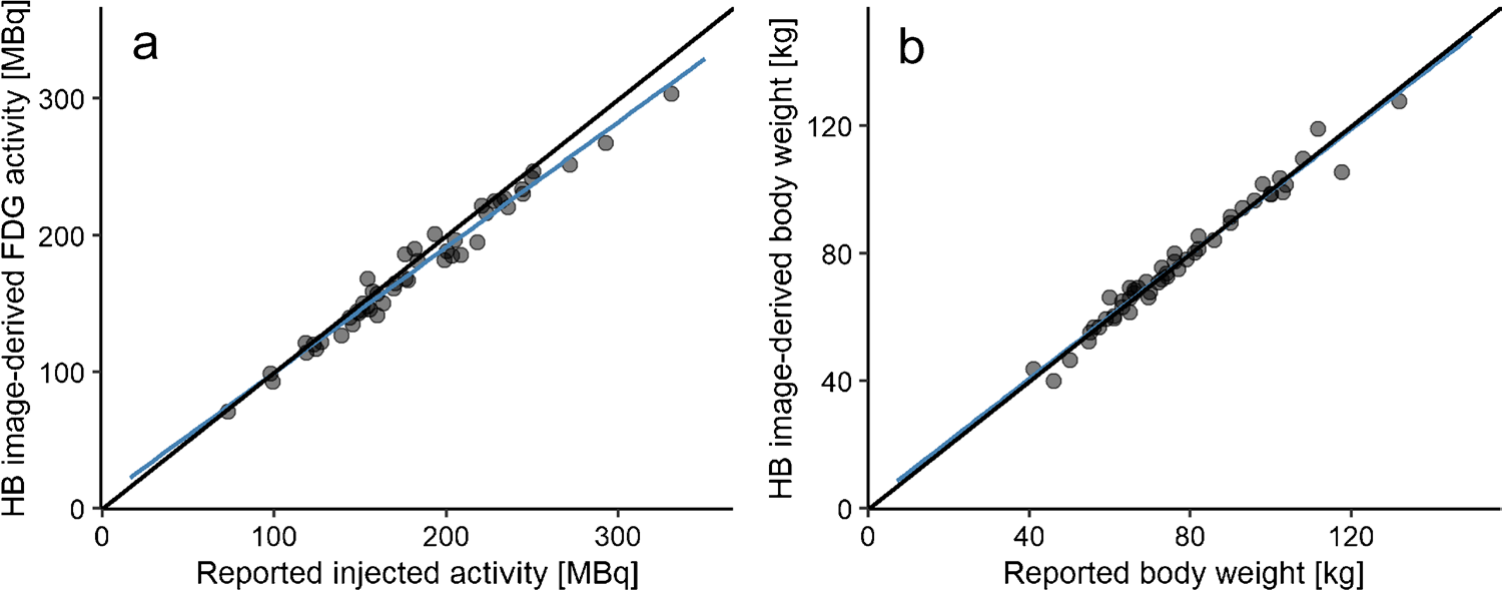
Correlation between reported and predicted injected activity (MBq) (**a**) and body weight (kg) (**b**) derived from half body (HB) ^18^F-FDG PET images acquired on a long-axial field-of-view (LAFOV) PET/CT system. The black line is the line of identity (LoI), and the blue line the fitted regression line

Scans with the aforementioned identified SUV errors (*n* = 7) were excluded to determine the correlation between reported values and HB image-derived values. After applying the regression fits (Eqs. 3 and 4), we found a strong correlation between reported injected activity and HB image-derived ^18^F-FDG activity (*r* = 0.988, *p* < 0.0001) and between reported body weight and HB image-derived body weight (*r* = 0.987, *p* < 0.0001). The correlations are illustrated in Fig. 3a, b, respectively. The relative absolute difference between reported ^18^F-FDG activity and predicted ^18^F-FDG activity was 4.9% ± 2.9%. The relative absolute difference between reported and predicted body weight was 3.1% ± 2.8%.

**Fig. 4:**
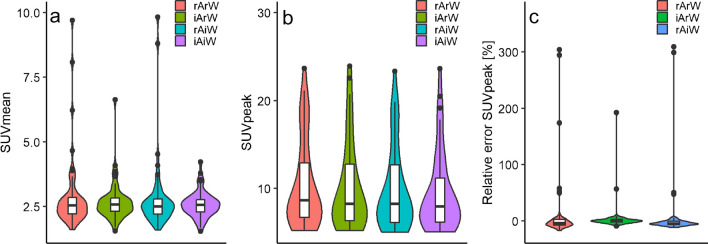
Liver SUVmean (**a**) and lesion SUVpeak (**b**) based on total body (TB) ^18^F-FDG PET images and the relative difference between the lesion SUVpeaks with the lesion SUVpeak based on image-derived activity and weight as reference (**c**). SUV is derived from reported (r) and image-derived (i) tracer activity (A) and patient weight (W). The central line of the boxplot represents the median, edges of the boxes are 25th and 75th percentiles

**Fig. 5:**
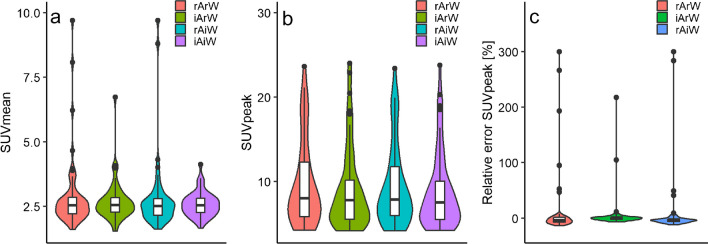
Liver SUVmean (**a**) and lesion SUVpeak (**b**) based on half body (HB) ^18^F-FDG PET images and the relative difference between the lesion SUVpeaks with the lesion SUVpeak based on image-derived activity and weight as reference (**c**). SUV is derived from reported (r) and image-derived (i) tracer activity (A) and patient weight (W). The central line of the boxplot represents the median, edges of the boxes are 25th and 75th percentiles

The effects of TB and HB image-derived ^18^F-FDG activity and body weight on liver SUVmean and lesion SUVpeak are illustrated in Figs. 4a, b, and 5a, b, respectively. For the TB and HB lesion SUVpeak, the percentage error between the SUVs, with the SUV based on image-derived activity and image-derived body weight as reference, is illustrated in Figs. 4c and 5c, respectively. Patients without ^18^F-FDG tumour uptake (*n* = 4) were excluded from the lesion SUVpeak analysis. ^18^F-FDG activity and body weight derived from TB scans reduced liver SUVmean and lesion SUVpeak variability compared with reported injected activity and patient weight (IQR 0.69 at reported activity and weight (rArW), IQR 0.50 at image-derived ^18^F-FDG activity and reported weight (iArW), IQR 0.63 at reported activity and image-derived body weight (rAiW), and IQR 0.47 image-derived activity and weight (iAiW)) (IQR 6.70 at rArW, IQR 4.69 at iArW, IQR 5.04 at rAiW, and IQR 4.56 at iAiW), respectively. HB image-derived ^18^F-FDG activity and body weight together with reported length also reduced liver SUVmean and lesion SUVpeak variability compared with reported injected activity and body weight (IQR 0.69 at rArW, IQR 0.56 at iArW, IQR 0.67 at rAiW, and IQR 0.53 at iAiW) (IQR 6.70 at rArW, IQR 4.64 at iArW, IQR 5.24 at rAiW, and IQR 4.52 at iAiW), respectively. Figures 4c and 5c illustrate the relative differences between SUV based on iAiW (reference) with the SUVs based on rArW, iArW, and rAiW. We found differences up to 309% between the SUV based on iAiW compared with SUV based on rArW. The use of TB and HB image-derived activity and body weight resolved those errors since the outliers were resolved when using either image-derived activity (iArW) or image-derived weight (rAiW).

### Validation of SAFOV dataset

**Fig. 6:**
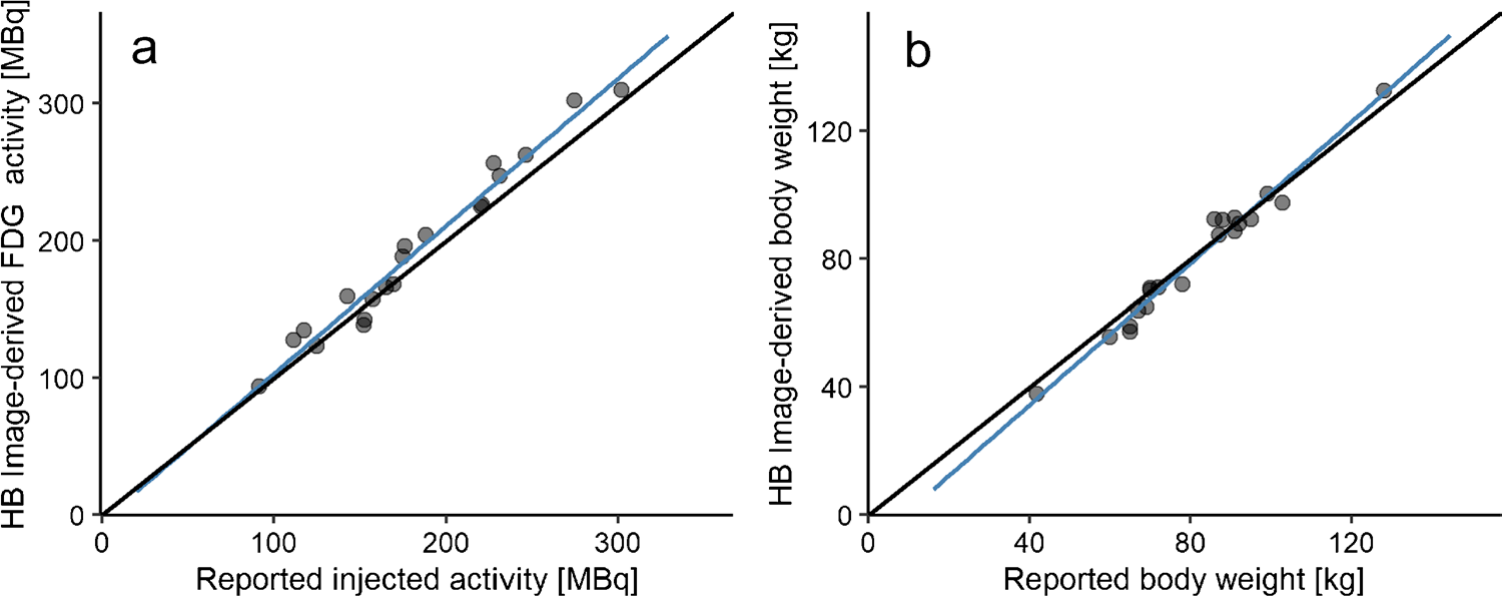
Correlation between reported and predicted injected activity (MBq) (**a**) and body weight (kg) (**b**) derived from half body (HB) ^18^F-FDG PET images acquired on a short-axial field-of-view (SAFOV) PET/CT system. The black line is the line of identity (LoI), and the blue line the fitted regression line

Twenty ^18^F-FDG PET scans were selected from the SAFOV PET/CT system. All image-derived values were corrected based on the earlier fitted HB regression equations (Eqs. 3 and 4) and thereafter compared with reported values. We found a strong correlation between reported injected activity and HB image-derived ^18^F-FDG activity (*r* = 0.984, *p* < 0.0001). A strong correlation was also found between reported body weight and HB image-derived body weight (*r* = 0.988, *p* < 0.0001). The correlations are illustrated in Fig. 6a, b, respectively. The relative absolute difference between reported and predicted ^18^F-FDG activity was 3.9% ± 3.7% and the relative absolute difference between reported and predicted body weight was 4.5% ± 3.4%.

## Discussion

This study aimed to examine if injected activity and body weight can be derived accurately from TB and HB ^18^F-FDG PET images and determined if image-derived ^18^F-FDG activity and body weight increased liver and lesion SUV precision. In addition, we aimed to assess if the regression fits based on a LAFOV PET/CT system were also applicable to HB ^18^F-FDG PET scans acquired on a SAFOV PET/CT system.

Our study showed that injected activity and body weight can be derived accurately within 4.8% ± 2.7% and 3.2% ± 2.7%, respectively, from TB ^18^F-FDG PET images and within 4.9% ± 2.9% and 3.1% ± 2.8%, respectively, from HB ^18^F-FDG scans on the LAFOV PET/CT scanner. Both TB and HB image-derived values improved liver SUV precision and corrected errors in lesion SUV. Furthermore, when using the regression fits from the LAFOV PET/CT system on HB ^18^F-FDG PET scans acquired on a SAFOV PET/CT system, injected activity and body weight can be derived within 6.7% ± 4.9% and 4.5% ± 3.4%, respectively.

The method used to derive image-based estimates is based on experimental regression of total image activity or weight versus reported activity or weight. We found that TB image-derived ^18^F-FDG activity underestimated reported injected activity by 14% and, consequently, our regression fit showed a scale factor of 1.14 (Eq. 1, *a* = 1.14). Approximately 10% can probably be attributed to activity loss due to emptying the bladder before scanning since ongoing dynamic studies show that around 10% of the total activity is accumulated in the bladder after 1 h (data not shown). The reported injected activity does not correct for this loss. Discrepancies in scanner calibration might explain the resulting percentage difference as the phantoms showed a scanner calibration accuracy of 3.4%. All these factors are corrected by using our regression-based approach. Note that we included scans ranging from the skull vertex to approximately the mid-thigh as part of our routine HB protocol in the LAFOV PET/CT.

The data demonstrated an accurate assessment of body weight and injected activity from both TB and HB PET scans acquired on a LAFOV PET/CT system. This suggests that activity and body weight can also be accurately derived from HB scans despite that the legs are not included in the scan trajectory. The regression fits can thus resolve the missing information that is not included in the HB scans. Deriving ^18^F-FDG activity and body weight might also effectively estimate missing injected activity and/or weight due to rigorous anonymisation of data in multi-centre studies. However, our HB image-derived method required reported length for accurate weight estimation. If reported length is also not available, new regression fits, that do not incorporate patient length, would be required to predict injected activity and body weight from the image-derived values. This may come at the cost of lower accuracy and precision if the scan trajectory is not dependent on patient length.

The equations to predict injected activity and body weight from HB ^18^F-FDG PET scans (Eqs. 7 and 8) were also able to estimate injected activity and body weight in HB ^18^F-FDG PET scans from a SAFOV PET/CT system. Image-derived weight from scans of a SAFOV PET/CT system showed a small increased variability compared with scans acquired on a LAFOV PET/CT system. However, differences were mostly seen in patients who were very light (42 kg) or heavy weighed (133 kg). The body weight of patients with an average weight (56 to 100 kg) was predicted accurately within 4.3%. It should be noted that variability of image-derived ^18^F-FDG activity increased when using scans from a SAFOV PET/CT system compared with a LAFOV PET/CT system. Image-derived injected activity slightly overestimated reported injected activity when using scans from a SAFOV PET/CT system. However, the overestimation still lies within the EARL standards and might be an effect of scanner calibration [19]. Therefore, we believe that our method is still suitable as quality control (QC) procedure in sites that only have SAFOV PET/CT systems and it can be used to detect outliers due to, e.g., data entry errors, use of wrong isotope settings, or incorrect clock synchronisation. These are not uncommon errors or outliers, and we also found these errors in our own dataset. It is important to note that the regression fits are only tested on a single SAFOV PET/CT system. We included only scans starting at the skull vertex and not scans ranging from the base of the skull to mid-thigh. Therefore, it is recommended to test if the regression parameters need adjustment in other sites and/or for other SAFOV systems before implementation in clinical practice. Yet, the proposed methodology or QC procedure seems to be equally applicable for both LAFOV and SAFOV PET/CT systems.

Image-derived values resolved errors and therefore reduced liver (and lesion) SUV variability compared with reported values. This bears importance since, nowadays, semi-quantitative uptake measurements are used more frequently in PET examinations. While individual errors might have a limited effect, multi-centre studies still show substantial errors in SUV results [20, 21]. Various reasons could be mentioned that affect the variability in reported SUV estimates and they were effectively resolved using image-derived estimates. Image-derived values resolved data entry errors in patient weight or injected activity, incorrect clock synchronisation between the PET/CT camera and dose calibrator clocks, and errors in decay correction due to selecting a wrong isotope. It also solves the problem of patients who cannot be weighed on a calibrated scale during their visit, e.g., if they are bedridden. Moreover, image-derived ^18^F-FDG activity and body weight can resolve errors in SUV estimation due to incorrect scanner calibration. Alternatively, normalising SUVs to a background region or organ, such as the liver or blood pool, has also been explored to reduce variability [22]. However, normalising all the data to a reference background, such as the liver, might not be a desirable solution since the reference organ can be affected by itself, and, e.g., liver uptake measurements can also be affected by biological factors such as chemotherapy, glucose levels, age, fasting, and delayed uptake time [16]. Therefore, adherence to performance standards, such as the EARL standards, and following ^18^F-FDG procedural guidelines is still recommended for ^18^F-FDG PET/CT imaging to generate reliable and reproducible lesion uptake measurements [1].

For this study, we choose to derive body weight from the ^18^F-FDG PET information, even though a CT-based method may be more accurate since adipose tissue has a lower density than muscles and other organs. However, the CT images would require additional corrections for the scanning table and fixation devices. Although CT information can be combined with the PET information to correct for this, we found that the current PET approach already performed very accurately over the entire range of observed patient weights. In future studies, it may be interesting to explore SUV accuracy when normalising it to CT-based lean body mass instead of body weight. Additionally, further exploring our proposed method with other radioisotopes, such as ^68^Ga or ^90^Y, may also be of great interest since other pharmaceuticals are also used in nuclear medicine.

In conclusion, we suggest that our proposed QC method should be used since it is important to detect errors in SUV caused by human errors, by clinical conditions preventing measuring patient weight or loss of essential information for SUV calculations due to rigorous anonymisation.

## Conclusion

In this paper, we studied if injected activity and patient weight can be derived from TB and HB scans on a LAFOV PET/CT system in order to mitigate SUV uncertainties and errors. We can conclude that ^18^F-FDG activity and body weight can be obtained from TB ^18^F-FDG PET scans within 4.8% ± 2.7% and 3.2% ± 2.7%, respectively, and from HB ^18^F-FDG PET scans within 4.9% ± 2.9% and 3.1% ± 2.8%, respectively. In addition, image-derived ^18^F-FDG activity and body weight improved liver SUV precision and corrected lesion SUV errors. Moreover, the proposed method was also able to accurately estimate injected activity and patient weight from PET images collected on a SAFOV PET/CT system. However, before using our proposed methods, they may require refinement of the suggested regression fits for each system or site individually. In conclusion, we recommend to use image-derived activity and patient weight as QC procedures to detect SUV errors and/or to improve SUV calculations.

## Data Availability

Data shown in the figures can be provided on reasonable request from the corresponding authors.
